# A Machine Learning Force Field for Bio-Macromolecular Modeling Based on Quantum Chemistry-Calculated Interaction Energy Datasets

**DOI:** 10.3390/bioengineering11010051

**Published:** 2024-01-03

**Authors:** Zhen-Xuan Fan, Sheng D. Chao

**Affiliations:** 1 Institute of Applied Mechanics, National Taiwan University, Taipei 106, Taiwan; r10543092@ntu.edu.tw; 2 Center for Quantum Science and Engineering, National Taiwan University, Taipei 106, Taiwan

**Keywords:** noncovalent interactions, machine learning force fields, symmetry-adapted perturbation theory, ab initio energy datasets, artificial intelligence

## Abstract

Accurate energy data from noncovalent interactions are essential for constructing force fields for molecular dynamics simulations of bio-macromolecular systems. There are two important practical issues in the construction of a reliable force field with the hope of balancing the desired chemical accuracy and working efficiency. One is to determine a suitable quantum chemistry level of theory for calculating interaction energies. The other is to use a suitable continuous energy function to model the quantum chemical energy data. For the first issue, we have recently calculated the intermolecular interaction energies using the SAPT0 level of theory, and we have systematically organized these energies into the ab initio SOFG-31 (homodimer) and SOFG-31-heterodimer datasets. In this work, we re-calculate these interaction energies by using the more advanced SAPT2 level of theory with a wider series of basis sets. Our purpose is to determine the SAPT level of theory proper for interaction energies with respect to the CCSD(T)/CBS benchmark chemical accuracy. Next, to utilize these energy datasets, we employ one of the well-developed machine learning techniques, called the CLIFF scheme, to construct a general-purpose force field for biomolecular dynamics simulations. Here we use the SOFG-31 dataset and the SOFG-31-heterodimer dataset as the training and test sets, respectively. Our results demonstrate that using the CLIFF scheme can reproduce a diverse range of dimeric interaction energy patterns with only a small training set. The overall errors for each SAPT energy component, as well as the SAPT total energy, are all well below the desired chemical accuracy of ~1 kcal/mol.

## 1. Introduction

Molecular dynamics (MD) simulations of chemical or material properties have been a very useful tool for understanding intricate experimental observations of bio-macromolecular systems, usually involving diverse spatial and temporal ranges. To obtain reliable MD simulation results, molecular modeling mainly relies on force field (FF) construction. The modeling of force fields usually starts with collecting observable data from distinct experiments. Together with statistical modeling methods, most traditional force field constructions employ a chemistry-based functional form, such as the famous Lennard-Jones potential, to model the empirical data available. This approach, usually called empirical force field (EFF) modeling, is favorable for obtaining a preliminary understanding of physical properties quickly. However, usually an EFF’s predictive power is weak. Not only the measured data input bears unknown empirical errors, but the chosen functional form may not be suitable for representing the intended force field. In practice, an EFF’s applicability is restricted to the boundaries of its original training set. Once far from the original training set, the predictive results become unreliable, and the conclusions are misleading. One way out of the first problem is to employ quantum chemistry-calculated ab initio energy data. These ab initio data can serve as a benchmark-level reference with minimum empirical information as the input.

In the past decade, we have witnessed an advancement in using quantum chemistry-calculated energy data to build potential energy surfaces (PESs) in the task of force field (FF) constructions [1,2,3,4,5,6,7,8,9,10,11]. In particular, it is now a routine calculation task to employ highly correlated ab initio methods, such as the second-order Møller–Plesset perturbation theory (MP2), to obtain accurate energy data for small molecular dimers with the number of atoms being less than about 50. Recently, these ab initio data have been collected and organized as easy-to-use datasets. These ab initio data can be used to calibrate less accurate but more efficient quantum mechanical methods, such as semiempirical methods. However, it still requires balancing accuracy and efficiency for obtaining meaningful predictions of the structures and energies of biomolecular or macromolecular systems. For this purpose, it is better to use the alternative symmetry-adapted perturbation theory (SAPT) for obtaining intermolecular interaction energies with satisfying chemical accuracy in a reasonable computational time [12,13,14,15,16,17,18,19,20,21,22]. Using the SAPT, one can directly calculate the interaction energies using the precalculated monomer properties. This method is favorable because it is free from basis set superposition errors (BSSEs) problems. Moreover, the theory divides the overall interaction energy into four theory-based terms: exchange, electrostatic, induction, and dispersion energy components. Thanks to these distinct features, the SAPT-calculated energy data are very useful in drug discovery and biomolecular recognition due to their acceptable accuracy and reasonable computational cost. Therefore, the SAPT method has been widely used in recent studies, with a great level of success in modeling biomolecular segments and motifs. In passing, we would like to mention that although these ab initio data are of very high accuracy, they are no substitute for reliable experimental data. The final judgment of the theory is still the experiment.

In our previous studies [23,24], we calculated the bonding structures and interaction energies for 31 homodimers of small organic functional groups dubbed the SOFG-31 dataset by using the MP2, CCSD(T), and the simplest SAPT0 level of theory. The SOFG-31 dataset consists of 31 monomers across 8 common classes, including 6 alkanes, 6 alkenes, 4 alkynes, 4 alcohols, 4 aldehydes, 3 ketones, 3 carboxylic acids, and 3 amides. Based on the SOFG-31 dataset, we also performed a parallel series of calculations to obtain the bonding structures and interaction energies for heterodimers selected from the combinations of monomers in the SOFG-31 dataset. This dataset is henceforth named the SOFG-31-heterodimer dataset. More specifically, the SOFG-31-heterodimer dataset contains 12 alkane-alkane (Aa-Aa), 16 alkane-alkene (Aa-Ae), 6 alkene-alkene (Ae-Ae), 16 alkane-alcohol (Aa-Ac), 16 alkane-aldehyde (Aa-Ad), 12 alkane-ketone (Aa-K), 16 alkene-alcohol (Ae-Ac), 16 alkene-aldehyde (Ae-Ad), 12 alkene-ketone (Ae-K), 12 alkane-carboxylic acid (Aa-Ca), 12 alkane-amide (Aa-Am), 12 alkene-carboxylic acid (Ae-Ca), 12 alkene-amide (Ae-Am), 6 alcohol-alcohol (Ac-Ac), 12 alcohol-carboxylic acid (Ac-Ca), 12 alcohol-amide (Ac-Am), 12 aldehyde-carboxylic acid (Ad-Ca), 12 aldehyde-amide (Ad-Am), and 15 binary complexes in the CAA-CAA set. These data are valuable thanks to their systematic organization according to the specific functional group types.

The second problem in force field modeling is how to model the ab initio data using a proper force function. This is the point where data analysis techniques can be very useful in this specific field of molecular modeling. The task of force field modeling over wide and diverse potential energy data, including both covalent and noncovalent interaction energies, usually involves a very complicated procedure and uses the special techniques of mathematical nonlinear regression. In practice, it is very difficult to uniquely determine a set of parameters with a given force function to represent the force field. Worse, when the dataset becomes larger, the corresponding parameter number also increases. Sometimes the parameter number is even greater than the data number, thus causing the overfitting problem. Recently, machine learning (ML) techniques have been used to solve this problem with a variable degree of success, and we would like to explore this interesting topic in traditional FF constructions. 

Recent artificial intelligence (AI)-generated methods, such as machine learning (ML) techniques, have been widely used in nearly all scientific applications [25,26,27,28,29,30,31,32,33,34,35,36,37,38,39]. A great number of ML algorithms, such as artificial neural networks (ANNs), kernel-ridge regression (KRR), and graph convolutional networks (GCNs), have been developed and tested [40,41,42,43]. These ML algorithms are particularly useful together with modern computer hardware structures, such as graphics processing units (GPUs). The AI-generated methods have rendered both successful stories and controversial examples. The promising success of the ML model can be attributed to the predictive ability of its algorithm to perform estimation on the unknown domains of system features with fast and quantitative nonlinear regression of the training data. Although in principle there exists a sound theoretical foundation for the ML algorithms [44,45,46,47,48,49,50], in practice most researchers reply to the more intuitive cycle of training, testing, and correcting. Therefore, it is very essential to prepare the input training data for ML modeling with the hope of finding a non-analytical function to represent the main features of a prepared dataset. To avoid immaterial outcomes, we should monitor the inherent black-box data propagation processes involved in the ML algorithm and judge the final results using human knowledge [51]. In sum, these ML algorithms can help a lot in solving many intricate problems in data modeling [52,53,54,55,56,57,58,59,60,61].

Recently, successful cases of utilizing these ML algorithms in macromolecular modeling, such as drug discovery, have been reported [62,63]. The purpose of molecular modeling is to well represent the noncovalent interactions (NCIs) involved in highly heterogeneous chemical, material, and biological environments [64,65,66]. These physical interactions are relatively weak as compared to usual chemical bonds but play a determining role in maintaining the equilibrium state of biomolecules such as lipids, proteins, and peptides in physiological conditions. The target object searched for in this kind of study is the unknown force functions for the NCIs, which are expected to be multidimensional. In this paper, we perform ML modeling on our previously constructed SOFG-31 and SOFG-31-heterodimer datasets. Our main purpose is to test a recently released ML algorithm called the CLIFF scheme [67]. In a previous study [68], we used a lower SAPT level of theory (SAPT0) to calculate the potential energy data and tested the ability of the CLIFF scheme to interpolate the datasets, with an emphasis on the possible overfitting problems. In this present paper, we would like to test the effect of changing the SAPT theory level on the predictive power and determine a minimum level of theory (SAPT2) for approaching the benchmark accuracy. The other parts of this paper are organized as follows: The main calculation results and due discussion are presented in Section 2. The dataset descriptions and methodological details are summarized in Section 3. The conclusions and perspectives are given in Section 4.

## 2. Results and Discussion

### 2.1. Preparation of the SOFG-31 Training Dataset

For the preparation of initial training datasets, two issues are important. One is to select a suitable quantum chemistry theory level for calculating the interaction energies in order to achieve the desired accuracy. The other is to sample the molecular structures in order to cover a wide range of chemical configuration spaces. One can first obtain the structures and energies with lower-accuracy methods. For example, one can perform a series of MD simulations with a standard EFF for a small system of molecules. The simulation would yield a set of randomly distributed structures and the corresponding interaction energies. These data bear no specific structural fingerprints, so they are often used together with a supervised ML algorithm to train the force field. With these preliminary structures, one can next systematically optimize the molecular geometry and calculate the energies using higher-accuracy methods at the optimized structures. Because the data contain important information about the structural features, they are usually used in a semi-supervised ML algorithm.

In this paper, we use the SOFG-31 dataset as the training dataset. The SOFG-31 dataset was home-made in our lab. The interaction energy data were calculated using a series of quantum chemistry methods, including the SAPT0 method. The SOFG-31 dataset contains 31 small molecules chosen from eight organic functional groups: alkanes (methane, ethane, propane, butane, pentane, hexane), alkenes (ethylene, propylene, butylene, pentylene), alkynes (ethyne, propylene, butylene, pentylene), alcohols (methanol, ethanol, propanol, butanol), aldehydes (formaldehyde, acetaldehyde, propanaldehyde, butanal), ketones (acetone, butanone, pentanone), carboxylic acids (formic acid, acetic acid, propanoic acid), and amides (formamide, acetamide, propenamide). All the dimers are optimized and found stable using the MP2 level of theory. In particular, we deliberately organize these data with respect to their functional group types. This is because, as mentioned above, we would like to employ human knowledge in training the force fields. Here a specific group bears its typical chemical features, which are well-known and well-collected in organic chemistry. As we have demonstrated in a previous study, the contribution propensity of the hydrogen bond attraction increases across the alkane group to the amide group. It is expected to utilize these known features in machine learning processes. In fact, we built the training set by taking into account the energy patterns for each group. The SOFG-31 dataset has been extended to include heterodimers selected from the SOFG-31 set and is henceforth called the SOFG-31-heterodimer dataset. In this paper, the SOFG-31 and the SOFG-31-heterodimer datasets are used as the training and test sets, respectively.

We have calculated the bonding structures and interaction energies for 31 homodimers of small organic functional groups, dubbed the SOFG-31 dataset, by using the MP2, CCSD(T), and the simplest SAPT0 level of theory. All the single-point energy calculations are performed at the MP2-optimized structures. We have tested the correlation between the SAPT0-calculated data and the benchmark CCSD(T) data and found that the SAPT0 level of theory has not reached a satisfying accuracy of about 1 kcal/mol. Notice that in the SI unit system, the unit for energy is kJ (=kcal/4.18), which is more often used in engineering studies. Therefore, we re-calculate the dimeric energies for both the SOFG-31 and SOFG-31-heterodimer datasets by using the more advanced SAPT2 level of theory. Table 1, Table 2 and Table 3 list the SAPT2-calculated interaction energies for the AaAeAy, AcAdK, and CAA subgroups, respectively. Notice that we have employed a wide series of basis sets, including the jun-cc-pVDZ (jDZ), jun-cc-pVTZ (jTZ), aug-cc-pVDZ (aDZ), and aug-cc-pVTZ (aTZ), in order to assess the basis set effects.

**Figure 1:**
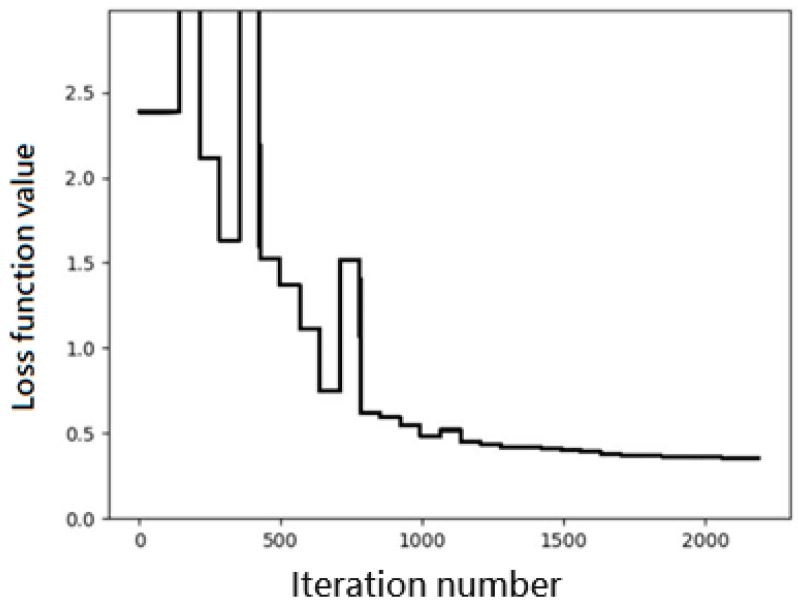
Convergence trend of the loss function during the iteration process. The unit is kcal/mol for the loss function value.

Next, we employ the SOFG-31 homodimer dataset calculated at the SAPT2/aTZ level of theory as our training set and utilize the CLIFF ML modeling scheme (see Section 3 for methodological details). Our purpose in this task is to determine the global parameters that gauge the intermolecular pairwise interactions separated into the four SAPT components. This is achieved by a nonlinear regression of the calculated energies with adjustments to the running parameter values. For the optimization task, we use the BFGS (Broyden–Fletcher–Goldfarb–Shanno) method with a multi-object loss function *L*. The mean square errors (MSEs) for the four SAPT component energies and the SAPT total energy are considered (Equation (1)), with the partition parameter γ = 0.4.

(1) $$L=(1-\gamma )\mathrm{MSE}(E_{total})+\gamma \sum_{i\in C}\mathrm{MSE}(E_{i})$$

where *C* represents the set of the four SAPT components. Figure 1 shows the convergence trend of the loss function during the iteration process, where the value of *L* is plotted versus the number of iterations. We observe a very quick convergence when the number of iterations exceeds about 700.

In Table 4, we show the optimized global parameters using the CLIFF scheme. Notice that parameters used in the CLIFF scheme involve two types. One is monomer-specific atomic parameters, such as atomic widths and multipole moments. The other is the dimeric global parameters shown here. The former atomic parameters have been fixed during the fitting processes. This means that we do not consider the atomic environmental changes due to bonding. To include the environmental effects, the CLIFF employs atom types. For example, the hydrogen atom types are defined based on the element of their bonding partner. With this set of global parameters, we have a first-version ML potential (see Section 3).

#### Using the SOFG-31 Training Set to Predict the SOFG-31-Heterodimer Test Set

**Figure 2:**
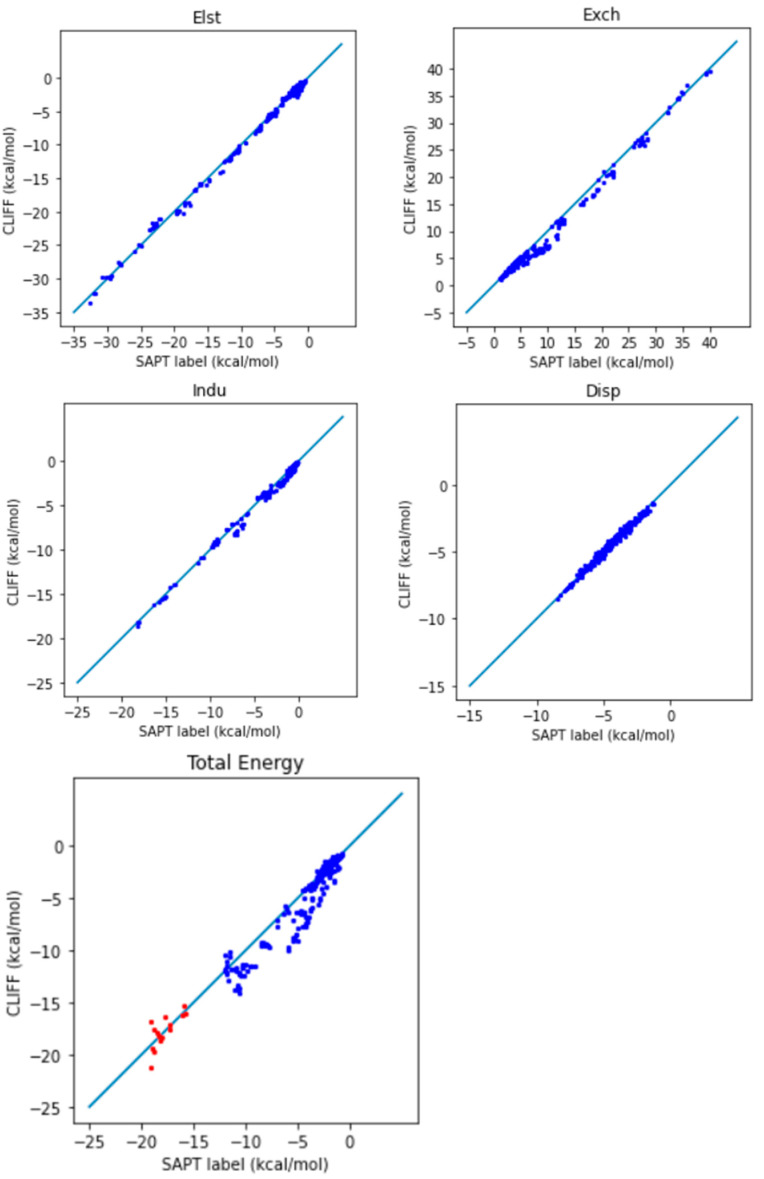
Correlation plots for the predicted and reference energies of the SOFG-31-heterodimer dataset using the SOFG-31 dataset as the training set. The non-hydrogen-bonded dimers are designated in blue color. The hydrogen-bonded dimers are designated in red color.

To evaluate the performance of this ML potential, where the global parameters are derived from the SOFG-31 training set, we now predict the energies of the dimers in the SOFG-31-heterodimer dataset. Table 5 lists the error measures between the predictive results and the reference SAPT2 energy data. Here, we use two length measures: the mean absolute error (MAE) and the root mean square error (RMSE). Both the SAPT component energies and the SAPT total energy have been tested. From Table 5, we see that both the MAE and the RMSE error measures are around the chemical accuracy of about 1 kcal/mol. Larger errors are associated with the exchange energy component, whose absolute energy values are also numerically larger. Figure 2 shows the correlation between the predicted energies and the reference SAPT2-calculated energies for the four SAPT energy components and the SAPT total energy. The deviation of the data distribution can be judged by the closeness of the points aligning along the diagonal reference line. We observe an overall well-aligned distribution of the predicted SAPT component energy data, albeit a biased underestimation of the exchange energy. Also notice that the van der Waals bounded dimers (i.e., those from the AaAeAy groups) exhibit a better symmetrical distribution. Again, the larger errors come from the exchange energy part.

For the total energy, the MAE using this ML potential is 0.932 kcal/mol, which is below the chemical accuracy of 1 kcal/mol. It is promising that the global parameters obtained by training the SOFG-31 dataset are suitable for predicting the energy data in the SOFG-31-heterodimer set. Though the training set contains only homodimers in equilibrium, the good predictive results for the heterodimers demand an explanation. The following facts may provide partial answers: The dimeric interaction energy is defined as a sum over paired atoms. Locally, the homodimer interactions include the pairwise information for the heterodimer interactions. That is, the homodimer interactions include interactions among different atom types, with a similar pattern to the heterodimers. It is thus understandable that we can use the ML potential derived from homodimer energies to predict or interpolate the heterodimer interactions.

**Figure 3:**
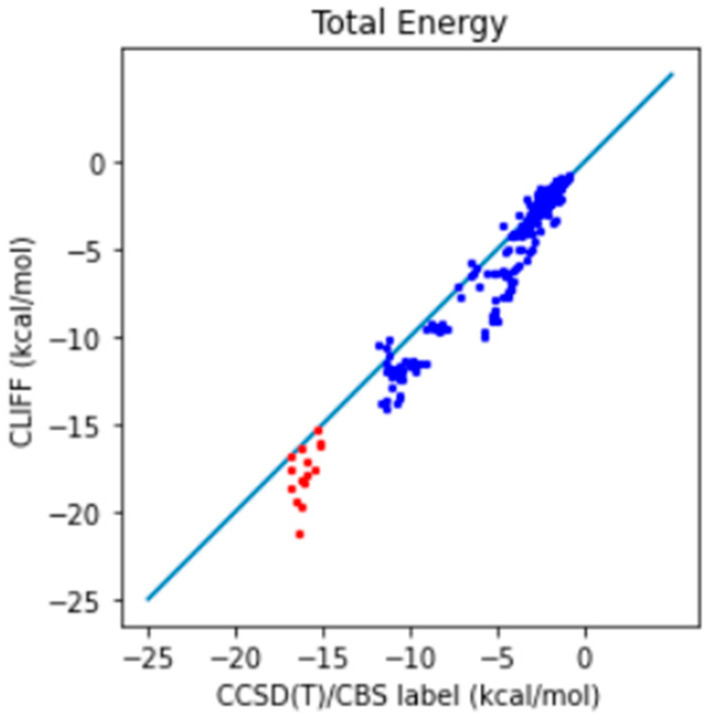
Correlation plot of the predictive and benchmark energies of the SOFG-31-heterodimer dataset using the SOFG-31 training set. The non-hydrogen-bonded dimers are designated in blue color. The hydrogen-bonded dimers are designated in red color.

In addition, we further compare the predictive results with the well-recognized gold standard CCSD(T)/CBS reference data. The correlation plot between the predictive energies and the reference data is shown in Figure 3. We see that the larger errors are associated with the hydrogen-bonded dimers (i.e., those from the CAA groups). For the total energy, the MAE is 0.991 (0.932) kcal/mol and the RMSE is 1.428 (1.380) kcal/mol, which should be compared with the previous results shown in the parentheses. These results show that using a higher level of theory is required for obtaining the benchmark energy data.

### 2.2. Employing the Dimer-31+9 as the Training Set

In order to make the predictive results closer to the benchmark energy data, we chose to include the heterodimers with large errors in the training set. For a specific subgroup, we gradually add the smaller heterodimers into the training set and perform the training. For each modification, we check whether the individual and the total errors are well controlled within 1 kcal/mol. In this way, we find it requires nine more heterodimers to obtain the best results. The nine dimers are Ethene-Methanol, Ethene-Ethanol, Ethene-Formic acid, Methanol-Formamide, Ethanol-Formamide, Formaldehyde-Formamide, Formaldehyde-Acetamide, Formaldehyde-Formic acid, and Formaldehyde-Acetic acid, and the new set is called the Dimer-31+9 training set. Next, the Dimer-31+9 dataset is used in the optimization process, as described in the last section. Table 6 lists the set of global parameters.

**Figure 4:**
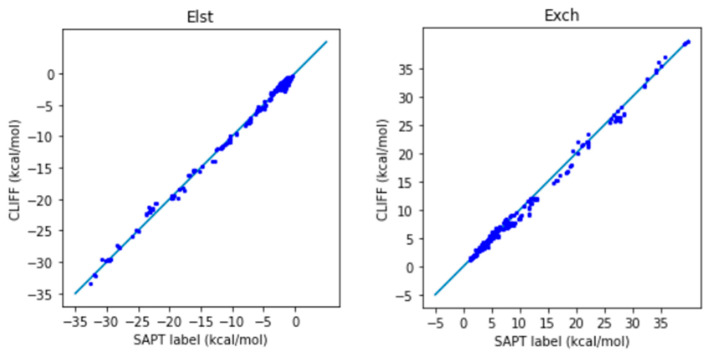

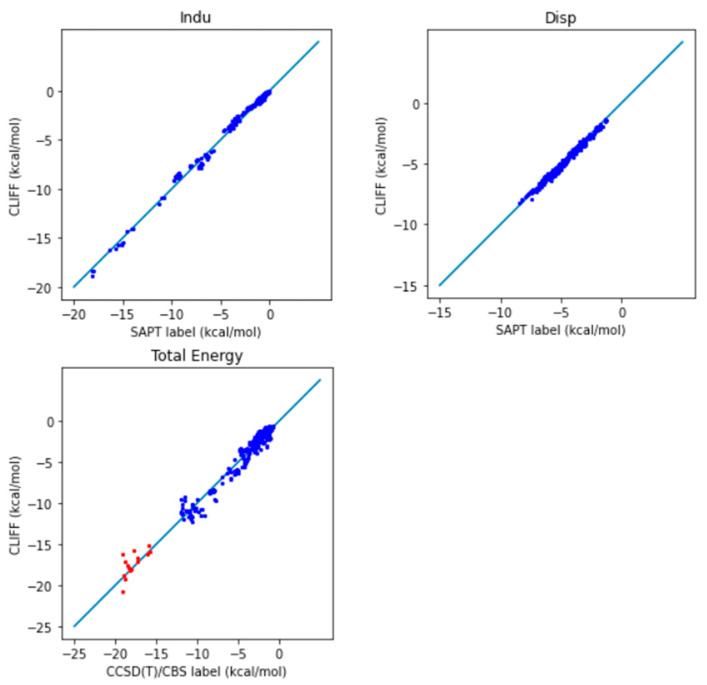
Correlation plots for the predicted and reference energies of the SOFG-31-heterodimer dataset using the Dimer-31+9 dataset as the training set. The non-hydrogen-bonded dimers are designated in blue color. The hydrogen-bonded dimers are designated in red color.

We now employ the global parameters based on the Dimer31+9 training set to test the SAPT energy data in the SOFG-31-heterodimer test set. Table 7 lists the error measures between the predictive results and the reference SAPT2 energy data. Again, two length measures, the MAE and the RMSE are used. We see clearly from Table 7 that both error measures are now well below 1 kcal/mol, which shows the good predictive ability of the ML potential. The correlation plots for the predicted and the SAPT2-calculated energies are shown in Figure 4 for the SAPT component and total energies, respectively. A closer distribution of points along the reference line indicates better prediction. We see that the predicted SAPT energy data are better aligned along the reference line. Also notice that the van der Waals-bounded dimers exhibit better alignment than the hydrogen-bonded dimers. The larger errors come from the exchange energy part. Our results clearly show that the MAE for the total SAPT energy is reduced from 0.932 kcal/mol to 0.605 kcal/mol, and the RMSE is reduced from 1.380 kcal/mol to 0.790 kcal/mol, which are significantly lower than the chemical standard. Here we demonstrate that adding a small set of heterodimers can greatly enhance the predictive power of the trained ML potential. 

**Figure 5:**
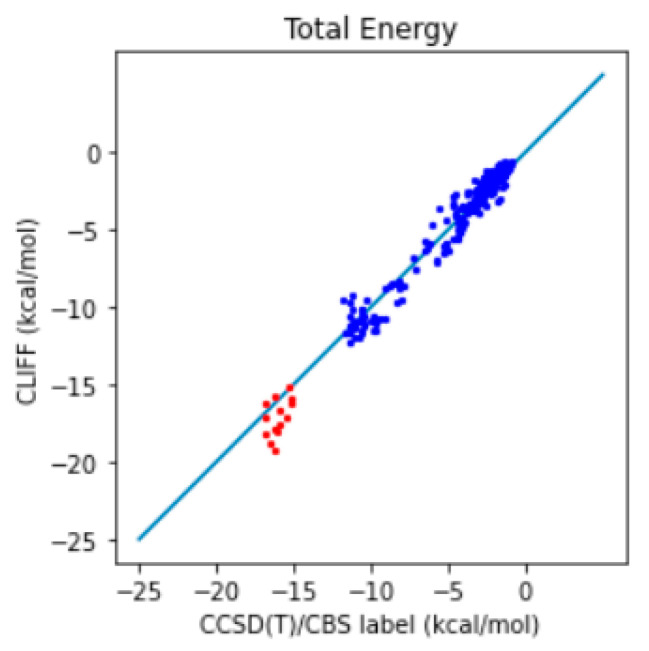
Correlation plot of the predictive and benchmark energies of the SOFG-31-heterodimer dataset using the Dimer-31+9 as the training set. The non-hydrogen-bonded dimers are designated in blue color. The hydrogen-bonded dimers are designated in red color.

In addition, we further compare the predictive results with the well-recognized gold standard CCSD(T)/CBS reference data. The correlation plot between the predictive energies and the reference data is shown in Figure 5. We see that the larger errors are associated with the hydrogen-bonded dimers (i.e., those from the CAA groups). For the total energy, the MAE is reduced from 0.991 to 0.643 kcal/mol and the RMSE from 1.428 to 0.858 kcal/mol. These results show that using a larger training set helps in obtaining the benchmark energy data. Because we have obtained a satisfying level of accuracy, we refer to the set of optimized global parameters as the CLIFF2 parameters, following the original CLIFF0 convention.

### 2.3. Using the CLIFF2 Parameters to Predict the Potential Energy Curves of the SOFG-31 and SOFG-31-Heterodimer Datasets

**Figure 6:**
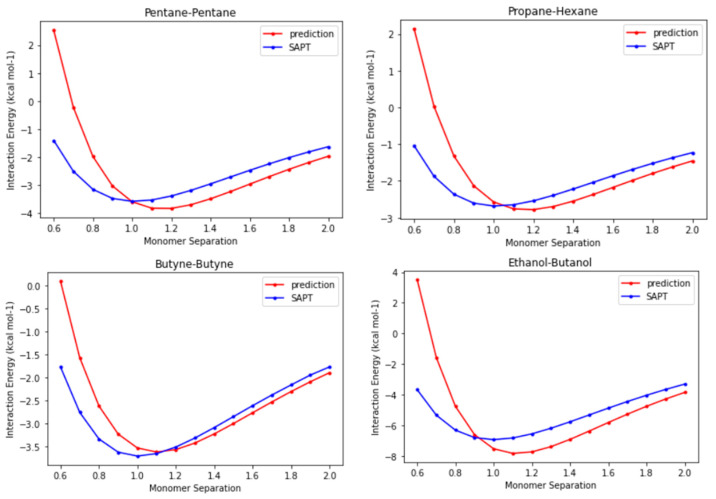
Comparison of the prediction energy curves with the destined SAPT energy curves for the pentane-pentane, the propane-hexane, the butyne-butyne, and the ethanol-butanol heterodimers. The monomer separation refers to the distance between the centers of mass of the involved monomers in the dimer and is normalized to the corresponding equilibrium distances, respectively.

Until this point in time, we have verified that the CLIFF2 parameters perform well in reproducing the interaction energies of dimers at equilibrium points, with the results even approaching the benchmark energies. Because the functional forms for the SAPT energy components are explicitly implemented in the CLIFF ML scheme, it is interesting to show the preliminary outlook for the whole potential energy curves. In Figure 6, we compare the prediction energy curves using the CLIFF2 parameters with the destined SAPT energy curves for four representative systems, namely, the pentane-pentane, the propane-hexane, the butyne-butyne, and the ethanol-butanol heterodimers. We plot the energy curves along the monomer separation, which is defined as the distance between the centers of mass of the involved monomers in the dimer. The distance unit is normalized to the equilibrium distance of the respective SAPT energy curve.

We first observe that the binding energies are well predicted with chemical accuracy. The equilibrium distances are not exactly reproduced, but they are all within 10% of the equilibrium distances. This is because in the CLIFF ML scheme, the geometry is unsupervised. For the far-distance side, the predicted energy curves are pretty precise if the equilibrium distances are shifted to the right places. However, the results for the short-distance side exhibit significant errors. The reason for this discrepancy can be partly attributed to the modeling formula for the exchange energy component (see Section 3). Nonetheless, it is noteworthy that using only energy data at equilibrium points proves effective in predicting energy curves, with correct trends for the overall profiles. This indicates that the energy models employed properly approximate the energy variation with distance. It requires further study to improve the prediction results for the molecular geometry and the short-distance side of the energy curves.

## 3. Materials and Methods

The energy data of the SOFG-31 dataset are arranged into 8 organic functional groups: alkanes, alkenes, alkynes, alcohols, aldehydes, ketones, amines, and carboxylic acids, resulting in 31 homodimers. The basis set superposition error (BSSE)-corrected super-molecule approach was used to calculate the interaction energies. The second-order Møller–Plesset perturbation theory (MP2) with the aug-cc-pV(D, T, Q)Z basis sets was used in geometry optimization. The benchmark interaction energies were calculated by the coupled cluster with single, double, and perturbative triple excitations at the complete basis set limit [CCSD(T)/CBS]. The groups of alkanes, alkenes, and alkynes are collectively called the AaAeAy group, while those of alcohols, aldehydes, and ketones are called the AcAdK group. The groups of carboxylic acids and amides are called the CAA group. The SOFG-31-heterodimer dataset is derived from selecting combinations of monomers from the SOFG-31 dataset. 

The total SAPT energy is decomposed into the following four components: exchange (Exch), electrostatic (Elst), dispersion (Disp), and induction (Indu) energies. The CLIFF scheme models these components using electronic density overlaps. Here we briefly summarize the mathematical equations for the four energy components, and the details should refer to the original CLIFF paper [67].

In this scheme, the exchange energy is described as the sum of all the repulsive interactions due to the overlapping electron densities between pairs of atoms.

(2) $$E_{exch}=\sum_{i\in A,j\in B}{K_{ij}}^{exch}S_{ij}$$

Here, the global parameters ${K_{i}}^{exch}$ , one for each atomic species, are determined through fitting to the SAPT-calculated exchange energies. The *S* matrices are calculated through the atomic valence widths, ${\;K}_{ij}^{exch}=K_{i}^{exch}K_{j}^{exch}$ for atom-typed parameters $K_{i}^{exch}$ .

(3) $$B_{ij}=\frac{1}{\sigma_{i}\sigma_{j}}$$

(4) $$S_{ij}=[\frac{1}{3}{\left(B_{ij}r_{ij}\right)}^{2}+B_{ij}r_{ij}+1]e^{-B_{ij}r_{ij}}$$

The electrostatic energy is modeled by the damped multipole electrostatic (DME) model, which considers interactions between the atomic nuclei for each atomic pair, between atomic nuclei and multipoles, and among multipoles [69].

(5) $$E_{elst}=\sum_{i\in A}\sum_{j\in B}\frac{Z_{i}Z_{j}}{r_{ij}}+{M_{i}}^{T}{T_{ij}}^{f_{1}}Z_{j}+Z_{i}{T_{ij}}^{f_{1}}M_{j}+{M_{i}}^{T}{T_{ij}}^{f_{2}}M_{j}$$

Each multipole matrix *M_i_* includes, in principle, all orders of multipoles, but in practice, the first non-vanishing multipoles are used. Here the *T* matrices are the damping interaction tensors among atomic nuclei and multipoles, respectively. The damping functions *f* are defined by the following equations:

(6) $$f_{1}(r_{ij})=1-e^{-{K_{i}}^{elst}r_{ij}}$$

(7) $$f_{2}(r_{ij})=1-\frac{{\left({K_{i}}^{elst}\right)}^{2}}{{\left({K_{i}}^{elst}\right)}^{2}-{\left({K_{j}}^{elst}\right)}^{2}}e^{-{K_{i}}^{elst}r_{ij}}-\frac{{\left({K_{j}}^{elst}\right)}^{2}}{{\left({K_{j}}^{elst}\right)}^{2}-{\left({K_{i}}^{elst}\right)}^{2}}e^{-{K_{j}}^{elst}r_{ij}}$$

where the global parameters ${K_{i}}^{elst}$ are determined by the ML modeling of the SAPT-calculated electrostatic energies. That is, the parameters are obtained by fitting the SAPT-calculated electrostatic energies.

The dispersion energy is modeled by the attractive interactions using the atomic polarization interacting with the involved electrons. Here, the popular Tang–Toennies model is used. Firstly, the coefficients for each atom pair are calculated using the following equations:

(8) $$C_{6,ij}=-\frac{2C_{6,i}C_{6,j}}{\frac{\alpha_{j}}{\alpha_{i}}C_{6,j}+\frac{\alpha_{i}}{\alpha_{j}}C_{6,i}}$$

(9) $$C_{6,i}=C_{6,i}^{free}{h_{i}}^{2},\alpha_{i}=\alpha_{i}^{free}h_{i}$$

Here *h_i_* designates the Hirshfeld ratio, defined as the ratio of the effective atoms-in-molecules (AIM) volume to the effective volume of the free atom, $\frac{V_{i}^{AIM}}{V_{i}^{free}}$ . $C_{6,i}$ stands for the monomer coefficients, and $\alpha_{i}$ represents the polarizability. The value $\alpha_{i}^{free}$ is determined by the free atomic density. To obtain $C_{8,ij}$ , we use

(10) $$C_{8,ij}=3C_{6,ij}\sqrt{Q_{i}Q_{j}},Q_{i}=\sqrt{Z_{i}}\frac{\langle r_{i}^{4}\rangle }{\langle r_{i}^{2}\rangle }$$

where $\langle r_{i}^{n}\rangle$ is the multipole expectation value from the atomic density. For $C_{10,ij}$ , we use

(11) $$C_{10,ij}=\frac{49}{40}\frac{C_{8,ij}^{2}}{C_{6,ij}}$$

Here the Tang–Toennies damping function is used.

(12) $$f_{n}=1-(\sum_{k=0}^{n}\frac{x_{ij}^{k}}{k!})e^{-x_{ij}},x_{ij}=B_{ij}r_{ij}+\frac{2B_{ij}^{2}+3B_{ij}}{{\left(B_{ij}r_{ij}\right)}^{2}+3B_{ij}r_{ij}+3}r_{ij}$$

Finally, we obtain the dispersion energy using the equation.

(13) $$E_{disp}=\sum_{i\in A}\sum_{j\in B}\left(\frac{C_{6,ij}}{r^{6}}f_{6}(r_{\mathrm{ij}})+K_{ij}^{disp}\sum_{n=8,10}\frac{C_{n,ij}}{r^{n}}f_{n}(r_{\mathrm{ij}})\right)$$

The global parameters $K^{disp}\;$ are determined by the ML modeling of the SAPT-calculated dispersion energies.

The induction energy is generated by the atomic polarization stimulated by the external electric field. Here, we use the Thole expression.

(14) $$E_{ind}=\sum_{i\in A}\sum_{j\in B}{\mu^{\prime }}_{i}T_{ij}M_{j}+K_{ij}^{indu}S_{ij}$$

where ${\mu^{\prime }}_{i}$ is the induced dipole, which can be derived from the iteration,

(15) $${\mu^{\prime }}_{i}(0)=\alpha_{i}\sum_{j\in B}T_{ij}M_{j}\;{\mu^{\prime }}_{i}(n+1)=(1-\omega ){\mu^{\prime }}_{i}(n)+\omega [{\mu^{\prime }}_{i}(0)+\alpha_{i}\sum_{\begin{array}{l} k\in A\cup B\\ k\neq i \end{array}}T_{ik}M_{k}]$$

where *k* sums over all other atoms (except the *i*-th atom) in the dimer and ω = 0.7. The interaction tensor, $T_{ij}$ , is used to smear the atomic charge distributions.

(16) $$f_{Thole}=\frac{3a}{4\pi }e^{-au^{3}},u=r_{ij}/{\left(\alpha_{i}\alpha_{j}\right)}^{\frac{1}{6}}$$

where a is the smearing coefficient set to 0.39. The global parameters $K^{indu}\;$ are determined with the ML modeling of the SAPT-calculated induction energies.

Because these formulas are written in pairwise sums, we need to obtain the electron densities of a monomer and distribute them to the constitutive atoms. The partitioning is calculated using the atoms-in-molecules (AIM) method. The AIM densities represent the atom-centered electronic clouds where local chemical surroundings have been considered. Notice that there are two groups of parameters. The atomic parameters include the atomic widths, the atomic multipoles, and the Hirshfield ratios. The global parameters include $K_{i}^{elst},\;K_{i}^{exch},{\;K}_{i}^{indu},\;and\;K_{i}^{disp}$ . The CLIFF ML scheme utilizes the AIM method to obtain the atomic multipoles, the atomic widths, and the Hirshfeld ratios from the AIM electron densities. To calculate the atomic reference densities, the PBE0/aug-cc-pV(D+d)Z method was used for the quantum chemistry calculations. Next, the atomic properties were determined with the minimum basis iterative stockholder (MBIS) partition method and the Hirshfeld routines, both implemented in the Horton (version 2.1.1, 2017) software. For the calculations of atomic multipoles, atomic widths, and Hirshfeld ratios, the CLIFF employed a KRR machine learning model for eight chemical elements (C, O, N, H, S, F, Cl, and Br). The training dataset contained molecules retrieved from the ChEMBL database. Firstly, the CLIFF selected a set of about 872,000 drug-like molecules. Second, the CLIFF classified molecules with respect to 5 to 12 heavy atoms. Finally, the CLIFF distinguished 8,138 molecules as core monomers to form dimers. These molecules are representatives of a wide and diverse range of drug-like compounds of medical interest.

## 4. Conclusions

We have performed a machine learning study on the recently proposed CLIFF kernel type modeling of intermolecular interactions for biomolecular dynamics simulations using our previously developed small organic functional group datasets, including the SOFG-31 and the SOFG-31-heterodimer sets. The training data were from the SOFG-31 dimer dataset and used in the CLIFF ML scheme. We built our ML potentials with the distinct features of these datasets, namely, the well-organized functional group types and the systematic inclusion of an analogous series of dimers in the training sets. Two tests were performed: (1) Training the SOFG-31 (homodimer) dataset to test the SOFG-31-heterodimer set, with an overall MAE of 0.991 kcal/mol. (2) Training the Dimer-31+9 dataset to test the SOFG-31-heterodimer set, with an overall MAE of 0.643 kcal/mol. Our results clearly show that it is possible to find a systematic construction rule for the training datasets, with which one can employ the CLIFF ML scheme to predict a wide range of geometric patterns and interaction energies. Therefore, it is promising to make the best use of well-tested machine learning techniques in force field modeling.

## Figures and Tables

**Table 1:** The SAPT2-calculated interaction energies (in kcal/mol) for the dimers in the AaAeAy groups. Basis sets include jun-cc-pVDZ (jDZ), jun-cc-pVTZ (jTZ), aug-cc-pVDZ (aDZ), and aug-cc-pVTZ (aTZ).

	SAPT2				CCSD(T)
	jDZ	jTZ	aDZ	aTZ	CBS
Methane	−0.123	−0.419	−0.420	−0.501	−0.530
Ethane	−0.498	−1.195	−1.117	−1.361	−1.388
Propane	−0.905	−1.824	−1.706	−2.024	−2.008
Butane	−1.447	−2.715	−2.549	−2.976	−2.819
Pentane	−1.991	−3.571	−3.362	−3.890	−3.662
Hexane	−2.557	−4.412	−4.174	−4.784	−4.505
Ethene	−0.524	−1.298	−1.179	−1.502	−1.478
Propene	−1.269	−2.289	−2.129	−2.491	−2.212
Butene	−1.327	−2.547	−2.350	−2.773	−2.323
Pentene	−1.770	−3.233	−3.014	−3.528	−3.170
Ethyne	−1.054	−1.504	−1.377	−1.628	−1.526
Propyne	−1.504	−2.416	−2.219	−2.678	−2.346
Butyne	−2.209	−3.713	−3.471	−4.054	−3.425
Pentyne	−2.941	−4.810	−4.497	−5.218	−4.450

**Table 2:** The SAPT2-calculated interaction energies (in kcal/mol) for the dimers in the AcAdK groups. Basis sets include jun-cc-pVDZ (jDZ), jun-cc-pVTZ (jTZ), aug-cc-pVDZ (aDZ), and aug-cc-pVTZ (aTZ).

	SAPT2				CCSD(T)
	jDZ	jTZ	aDZ	aTZ	CBS
Methanol	−4.716	−5.421	−5.049	−5.565	−5.848
Ethanol	−5.232	−6.492	−6.034	−6.708	−6.805
Propanol	−5.445	−6.805	−6.327	−7.067	−7.155
Butanol	−6.004	−7.412	−6.972	−7.687	−7.297
Formaldehyde	−2.751	−4.126	−3.819	−4.418	−4.602
Acetaldehyde	−3.301	−4.563	−4.351	−4.806	−5.168
Propanal	−3.564	−5.243	−4.980	−5.562	−5.448
Butanal	−4.023	−5.627	−5.394	−5.907	−5.734
Acetone	−4.907	−6.806	−6.446	−7.145	−6.903
Butanone	−5.168	−7.076	−6.772	−7.407	−7.141
Pentanone	−5.538	−7.553	−7.239	−7.894	−7.367

**Table 3:** The SAPT2-calculated interaction energies (in kcal/mol) for the dimers in the CAA groups. Basis sets include jun-cc-pVDZ (jDZ), jun-cc-pVTZ (jTZ), aug-cc-pVDZ (aDZ), and aug-cc-pVTZ (aTZ).

	SAPT2				CCSD(T)
	jDZ	jTZ	aDZ	aTZ	CBS
Formic acid	−14.967	−18.420	−16.978	−18.766	−18.733
Acetic acid	−15.845	−19.166	−17.683	−19.485	−19.317
Propanoic acid	−17.063	−20.572	−19.003	−20.894	−20.292
Formamide	−12.780	−15.439	−14.557	−15.769	−16.077
Acetamide	−13.332	−15.921	−14.987	−16.234	−16.274
Propanamide	−13.694	−16.294	−15.351	−16.612	−16.154

**Table 4:** The CLIFF atom types and global parameters with the training set SOFG-31.

	$K^{elst}$	$K^{exch}$	$K^{indu}$	$K^{disp}$
HC	3.644	0.982	0.144	0.003
HN	2.903	0.978	0.678	0.046
HO	2.683	0.741	0.734	0.018
C4	3.229	2.261	0.676	0.214
C3	3.336	2.476	1.616	0.377
C2	3.119	2.589	0.938	0.667
N3	3.358	4.333	1.877	0.079
O1	3.885	4.676	1.530	0.660
O2	4.752	5.405	1.025	0.107

**Table 5:** Using the SOFG-31 trained ML potential to predict energy data in the SOFG-31-heterodimer set. (Energy in kcal/mol, 1 kcal = 4.18 kJ/mol).

	MAE	RMSE
Elst	0.326	0.455
Exch	0.749	1.104
Indu	0.273	0.142
Disp	0.158	0.204
Total	0.932	1.380

**Table 6:** The CLIFF atom types and global parameters with the training set Dimer-31+9.

	$K^{elst}$	$K^{exch}$	$K^{indu}$	$K^{disp}$
HC	4.750	1.061	0.003	0.162
HN	2.647	0.965	0.444	0.153
HO	2.632	0.905	0.427	0.059
C4	2.999	2.065	0.787	0.060
C3	3.323	2.979	1.265	0.740
C2	3.162	2.575	1.186	0.674
N3	4.111	6.546	0.924	0.019
O1	3.977	4.016	2.598	0.158
O2	5.115	4.656	1.509	0.057

**Table 7:** Using the Dimer-31+9 set to predict the SOFG-31-heterodimer results (energy in kcal/mol, 1 kcal = 4.18 kJ/mol).

Dimer-31+9(fit)	MAE	RMSE
Elst	0.340	0.488
Exch	0.550	0.730
Indu	0.254	0.332
Disp	0.169	0.214
Total	0.605	0.790

## Data Availability

The data that supports the findings of this study are available within the article and can be obtained from the authors.
